# Mining protein interactomes to improve their reliability and support the advancement of network medicine

**DOI:** 10.3389/fgene.2015.00296

**Published:** 2015-09-23

**Authors:** Gregorio Alanis-Lobato

**Affiliations:** ^1^Faculty of Biology, Institute of Molecular Biology, Johannes Gutenberg University of MainzMainz, Germany; ^2^Integrative Systems Biology Lab, Biological and Environmental Sciences and Engineering Division, King Abdullah University of Science and TechnologyThuwal, Saudi Arabia

**Keywords:** interactome, proteome, network, reliability, prediction, medicine, disease, pathogenesis

## Abstract

High-throughput detection of protein interactions has had a major impact in our understanding of the intricate molecular machinery underlying the living cell, and has permitted the construction of very large protein interactomes. The protein networks that are currently available are incomplete and a significant percentage of their interactions are false positives. Fortunately, the structural properties observed in good quality social or technological networks are also present in biological systems. This has encouraged the development of tools, to improve the reliability of protein networks and predict new interactions based merely on the topological characteristics of their components. Since diseases are rarely caused by the malfunction of a single protein, having a more complete and reliable interactome is crucial in order to identify groups of inter-related proteins involved in disease etiology. These system components can then be targeted with minimal collateral damage. In this article, an important number of network mining tools is reviewed, together with resources from which reliable protein interactomes can be constructed. In addition to the review, a few representative examples of how molecular and clinical data can be integrated to deepen our understanding of pathogenesis are discussed.

## 1. Introduction

The existence of living cells is not possible without organized and coordinated communication between proteins. Failure of the control mechanisms that underlie these delicate relationships can lead to disease or even death (Lesk, 2007). This highlights that the study of the complex network of interactions between proteins is crucial to improve our understanding of the intricate mechanisms that make life possible (Lesk, 2007; Loscalzo and Barabasi, 2011). To ease the analysis of this involved biological machine, it is commonly represented as a network of nodes, linked to each other if there is evidence of their physical or functional relationship.

**Figure 1:**
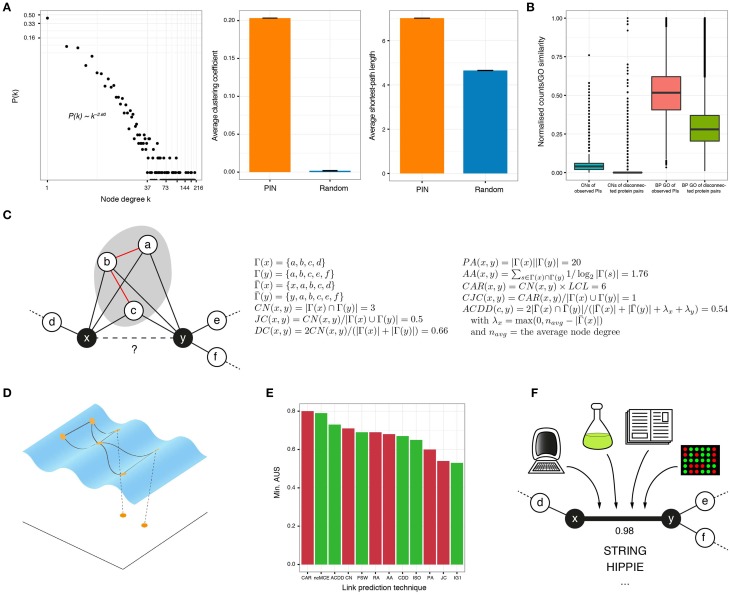
**(A)** In protein interactomes, only a few proteins, known as hubs, have a high number of interactors (node degree) and the rest interact only with a small number of proteins (left). In addition, PINs are highly clustered (middle) and every protein is easily reachable from anywhere in the network (right), compared to graphs with the same number of randomly linked nodes. **(B)** The number of common neighbors (normalized by the maximum) and the gene ontology (GO) similarity (biological process or BP shown) of protein interactions is higher than for disconnected protein pairs in the observed network. **(C)** The goal of neighborhood-based link prediction and reliability assessment is to assign a likelihood score to the observed or potential interaction between two proteins x and y. The formulae for representative link predictors are listed and applied to the toy network on the left. Γ(*x*) is the set of neighbors of node x, $\bar{\Gamma }\left(x\right)$ is the same set but including x and the local community links (LCL) are highlighted in red. **(D)** There is compelling evidence that complex networks, like PINs, lie on low-dimensional manifolds embedded in high-dimensional space. When protein networks are mapped to low dimensions, good candidate interactions lie in close proximity. **(E)** The link prediction performance of several of the topological techniques discussed in this review, measured by the minimum Area Under the Sparsification curve (AUS) amongst four networks (for details of these datasets, see Cannistraci et al., 2013b). Red bars correspond to methods proposed for networks in general and green bars to methods proposed for bio-networks. **(F)** High-quality PI resources, like STRING or HIPPIE, assign a confidence score to each of their reported interactions, based on the different evidence sources supporting them.

Today we have access to vast Protein Interaction Networks (PINs) from different organisms, due to high-throughput experimental techniques that are often an improved variation of yeast-two-hybrid screenings, or of co-immunoprecipitation followed by mass spectrometry (Vidal et al., 2011). Nevertheless, these networks are incomplete and contain a significant number of false positive interactions (Kuchaiev et al., 2009). However, it is fortunate that their structural properties are not different from those observed in good quality social or technological networks (Albert and Barabási, 2002; Liu et al., 2011; Cannistraci et al., 2013a) (Figure 1A). These topological similarities have prompted the development of tools, based on node-connectivity properties, aimed at improving the reliability and completeness of complex networks (Cannistraci et al., 2013a).

The reliability indices and predictions resulting from the application of these methods can be integrated with other sources of high-quality protein interactions (PIs). With these, one can construct reliable PINs that, together with clinical and genetic data, represent the fundamental pieces of information used in the emerging field of network medicine (Barabási et al., 2011; Loscalzo and Barabasi, 2011).

## 2. Topological reliability assessment and prediction of protein interactions

The observable network topologies of biological systems are not complete and contain spurious interactions. In addition, the mechanisms that lead two proteins to interact are not fully understood yet. As a consequence, traditional machine learning algorithms cannot be easily applied to PINs. Not only is the definition of features to discriminate between interacting and non-interacting proteins a challenging task, but also the construction of positive and negative sets of interactions to train these algorithms. For example, two unlinked proteins in the observable network cannot be considered as part of the negative set: it could very well be that they are disconnected due to experimental constraints that prevented scientists from observing their interaction. Alternatively it could be that, two linked proteins represent a false positive that is part of the dataset because one of the interactors is, for example, a sticky protein (Saito et al., 2002).

In this context, the assignment of likelihood scores to connected and disconnected pairs of proteins, on the mere basis of the observable network topology, is a convenient means to improve the degree of confidence and completeness of PINs (Cannistraci et al., 2013a). Although reliability assessment of PIs deals with connected proteins pairs and PI prediction with disconnected pairs, the methods used for one or the other are the same. The following subsections account for the most important techniques to perform these functions. A more in-depth description of these approaches can be found in, for example, Lü and Zhou (2011).

### 2.1. Neighborhood-based techniques

In 2001, Newman found that the relative probability of collaboration between scientists increases with their number of common acquaintances (Newman, 2001). Figure 1B shows that this is also applicable to PINs: the number of common neighbors (CNs) is higher for connected protein pairs than for disconnected ones, in a high quality human interactome. This inspired the creation of the CN index, which assigns high likelihood scores to protein pairs with many CNs.

Newman's findings triggered the development of a myriad of neighborhood-based approaches (Lü and Zhou, 2011). Some of them are only normalizations of CN, like Jaccard's index (Jaccard, 1912) or the Dice Similarity (Dice, 1945), but others really depart from it. For example, Preferential Attachment (PA) (Newman, 2001) is the product between the number of neighbors of the two nodes being analyzed, and Adamic and Adar (2003) and Resource Allocation (Zhou et al., 2009) assign higher likelihood scores to node pairs whose CNs do not interact with other components. Other indices, like Local Path (Lü et al., 2009) or Katz (Katz, 1953), not only take the number of CNs into account but also the neighbors of these CNs and so on, up to a user-specified depth.

In 2013, Cannistraci and colleagues introduced a paradigm shift in topological link prediction, by noting that the presence of a tightly connected set of CNs increases the probability of interaction between non-adjacent nodes (Cannistraci et al., 2013a). Thus, they introduced a family of neighborhood-based approaches by changing the formulation of popular techniques with the inclusion of the number of links between CNs. The simplest example is the so-called Cannistraci-Alanis-Ravasi index (CAR) that multiples this number by CN.

Although the above mentioned techniques can be applied to PINs, they were formulated for networks in general and do not consider any particular biological assumption. The pioneers of PI reliability assessment and prediction are Saito and colleagues. In 2002, after observing that the partners of sticky proteins and self-activators do not interact with anything else in PINs, they proposed the Interaction Generality index (IG1), which assigns low reliability scores to protein pairs whose neighbors have very few partners (Saito et al., 2002). They later introduced the IG2, which postulates that closed-loop motifs are indicative of PIs (Saito et al., 2003).

Another two indices put forward in the context of protein interactomes are the Interaction Reliability by Alternative Paths index (IRAP) and its successor IRAP^*^ (Chen et al., 2006b). According to these indices, the likelihood that two proteins interact increases if there is a large number of alternative network paths through which they can communicate. Unfortunately, these techniques, together with IG2, are computationally demanding, which prompted the development of more efficient and accurate methods (Chen et al., 2006a) such as the Functional Similarity Weight (FSW) and the Adjusted Czekanowski-Dice Dissimilarity (Chua et al., 2006; Liu et al., 2009; Alanis-Lobato et al., 2013). These approaches are interesting because they bet for a lenient integration of the CN and PA indices: protein pairs with lots of common interactors are good candidate PIs, but if one of the two proteins has very few partners, the confidence score is penalized.

All the afore-mentioned techniques represent, in general, an efficient and accurate way to identify protein pairs that are good candidates for interaction (see the formulation of some of them and their application to a toy example in Figure 1C). However, they all strongly depend on topological information to work properly. As a consequence, they perform poorly when applied to very sparse networks, like the PINs of non-model or poorly annotated organisms (You et al., 2010).

### 2.2. Maximum likelihood techniques

Maximum likelihood approaches, introduced mainly for link prediction, rely on the underlying community structure of complex networks. In the Hierarchical Random Graph (Clauset et al., 2008), the space of all possible dendrograms of a network is searched to get the ones that best fit its hierarchical structure. Non-adjacent pairs of nodes that have high average probability of being connected within these dendrograms represent good candidates for interaction. In the Stochastic Block Model (Guimerà and Sales-Pardo, 2009), in which a network is partitioned into groups, the probability that two nodes are connected depends on the groups to which they belong. An important issue with these approaches is that they are computationally expensive and not parameter-free (Lü and Zhou, 2011).

### 2.3. Network embedding techniques

Data analysts are regularly faced with the problem of finding meaningful low-dimensional representations of high-dimensional data. Algorithms such as Multidimensional Scaling or Principal Component Analysis embed data to low dimensions by preserving inter-sample distances or covariances but, if the dataset under study contains non-linear structure, they fail to provide useful mappings (Tenenbaum et al., 2000). To solve this issue, non-linear dimensionality reduction algorithms, such as Isometric Feature Mapping (ISOMAP), are commonly employed. Under the hypothesis that the biological features that lead to a PI are complex and non-linear, one could assume that PINs are shaped over a manifold embedded in a high-dimensional space, where interacting proteins are geometrically close to each other and disconnected pairs are far apart (Kuchaiev et al., 2009; You et al., 2010; Cannistraci et al., 2013a). This highlights that if a reasonable measure of dissimilarity between proteins is established, a pairwise dissimilarity matrix can be constructed and used to reveal the low-dimensional geometry of the analyzed network. Good candidates for interaction are finally determined via closeness relationships in the reduced space (Figure 1D).

Nataša Pržulj and her colleagues are pioneers in the modeling of PINs with geometric graphs. Their computational experiments show close matches between important topological properties of PINs and geometric random graphs (Przulj et al., 2004). Their results support the hypothesis that PINs do have an underlying geometric structure. These conclusions resulted from the embedding of networks to low dimensions, using the shortest-paths between nodes as dissimilarity and investigating whether proteins pairs that map close to each other are indeed more likely to interact (Higham et al., 2008; Kuchaiev et al., 2009). In 2010, You and co-workers extended this idea with the application of FSW to the PIN after embedding, with the aim to refine the identification of candidate PIs (You et al., 2010).

Around the same time period, a group of physicists and network scientists were independently developing a framework to model complex networks, resting on the assumption that a hidden metric space underlies them and shapes their topology (Boguñá et al., 2009). Contrary to Pržulj and You, who map PINs to a Euclidean space, this group's hypothesis is that complex networks respect the rules of hyperbolic spaces (Krioukov et al., 2010, 2012). This choice is reasonable: trees (subgraphs touching all network nodes without cycles), which abstract the skeleton or hierarchy of complex networks, need an exponential amount of space to branch [the total number of nodes at depth *d* in a *b*-ary tree is (*b*^*d*+1^ − 1)/(*b* − 1)] and only hyperbolic spaces expand exponentially, providing enough space for a complex network to grow (Krioukov et al., 2010). This premise evolved into a model able to produce scale-free and strongly clustered networks, by simply distributing nodes at random in hyperbolic space and connecting those that are hyperbolically close to each other (Papadopoulos et al., 2012). In addition, the fact that two nodes are connected in a real network correlates strikingly well with short hyperbolic distances between them (Krioukov et al., 2010; Papadopoulos et al., 2012). These results confirm that complex networks, like PINs, do possess an intrinsic organization shaped by geometric principles that agree well with hyperbolic ones. However, current algorithms to map networks to hyperbolic space depend on a Metropolis-Hastings algorithm that requires some manual intervention to converge in a reasonable amount of time (Papadopoulos et al., 2012). More computationally efficient methods are currently under development.

Finally, in the non-centered Minimum Curvilinear Embedding (ncMCE), a technique that has been successfully applied in different fields (Alanis-Lobato et al., 2015), the Minimum Spanning Tree (MST) is extracted from the network under scrutiny to construct a matrix of pairwise distances between nodes over the MST. The network is then projected to low-dimensions by singular value decomposition of this matrix and, in contrast to previous approaches, that assign likelihood scores by directly measuring Euclidean distances between node pairs (Kuchaiev et al., 2009; You et al., 2010), in ncMCE the network is reconstructed in the reduced space so that its edges are weighted by the distances between connected nodes. Likelihood scores are then the shortest-paths between nodes in this low-dimensionally projected, weighted network (Cannistraci et al., 2013b). It is not surprising that this technique achieves a remarkable performance in the prediction of PIs: measuring distances between proteins over the MST, corresponds to navigating one of the discrete representations of the hyperbolic geometry underlying the network under study. As previously mentioned, hyperbolic spaces are smooth versions of the trees abstracting the hierarchy of PINs (Krioukov et al., 2010).

### 2.4. General framework for measuring the effectiveness of these techniques

In order to benchmark the accuracy of a link prediction technique, the following framework is commonly employed:

1. Remove *L* randomly selected PIs from the observable network topology.
2. Assign confidence scores to disconnected protein pairs in the pruned network with a topological technique and sort them decreasingly (best candidate interactions positioned at the top of this list).
3. Take *L* protein pairs from the top of the sorted list and compute the proportion present in the set of interactions removed in 1. This is a measure of the technique's *precision*.
4. Repeat steps 1–3 *t* times, removing different sets of randomly selected PIs.
5. Repeat steps 1–4 removing 2*L*, 3*L*, etc. interactions, up to the point where the network loses connectivity. This allows for the construction of a sparsification curve (SC), whose points are the mean precisions of the technique applied at each sparsification level.

This evaluation depicts the ability of a topological approach to predict accurately under the presence of less and less network information. Nonetheless, it has an intrinsic problem because, as discussed above, some of the candidate interactions with high confidence scores may not be part of the randomly removed set of PIs. However, they may represent good candidates that current technologies cannot measure. Moreover, members of the removed set of links may be false positives that good link predictors are correctly discarding by giving them low scores. Subsequently, researchers have opted for using Gene Ontology (GO) similarities (Yu et al., 2010) to discriminate between good and bad candidate PIs. This is based on the *guilt-by-association* principle (Oliver, 2000), which states that if two proteins are involved in similar bio-processes, they are more likely to interact (see Figure 1B). Although Resnik's index (Resnik, 1999) is the prevailing GO similarity, Wang's index is worth mentioning because it was formulated specifically for the GO (Wang et al., 2007). Another interesting method improves GO similarities by considering the inherent uncertainty originating from the GO incompleteness (Yang et al., 2012).

Figure 1E presents the minimum area under the SC for most of the topological techniques described in this section, when they are applied to four yeast networks for the link prediction task (Cannistraci et al., 2013a,b). This figure depicts the robustness of each technique, as their worst performance is exposed. Despite the good results of some of these methods, there is still room for improvement, and development of approaches that consider the scale-free structure and geometry of PINs remain active subjects of research (Papadopoulos et al., 2012; Zhu et al., 2013).

## 3. Resources for high confidence protein interactions

Proteins with a high likelihood to interact can considerably reduce the universe of possible pairs to test in the lab and guide wet-lab validations. These interactions can then be integrated with available repositories of high-quality PIs that attach confidence scores to each reported interaction (see Figure 1F). One of such resources is the Search Tool for the Retrieval of Interacting Genes (STRING), which provides a combined score that indicates higher confidence when more than one source of evidence supports an interaction (Szklarczyk et al., 2011). STRING evidence sources include computational associations (neighborhood-based, co-occurrence, co-expression, text mining), high-throughput experiments, other databases, and interactions identified in other organisms. The current version of STRING (available at http://string-db.org) provides an interactive network viewer and access to interactions between almost 10 million proteins, from more than 2000 organisms (Szklarczyk et al., 2015).

The Human Integrated Protein-Protein Interaction rEference (HIPPIE) retrieves interactions from major expert-curated databases and calculates a score for each PI, reflecting its combined experimental evidence. This score is a function of the number of studies supporting the interaction, the quality of the experimental techniques used to measure it and the number of organisms in which it is present (Schaefer et al., 2012). In HIPPIE (http://cbdm-01.zdv.uni-mainz.de/~mschaefer/hippie/), one can query the interactors of a protein or a set of proteins and explore the resulting network in an interactive viewer. Furthermore, the results can be filtered by PI type, tissue, functions, directionality and inhibitory/activating effect (Schaefer et al., 2013).

Another worth-mentioning resource is INstruct (http://instruct.yulab.org/). It collects interactions from eight major expert-curated databases and filters out low-quality PIs, to keep only those supported by domain-domain interactions obtained from co-crystal structures (Wang et al., 2012; Meyer et al., 2013). INstruct provides a web-based interface to query its extremely high-quality PINs for 7 different species. The network properties depicted in Figures 1A–C correspond to the INstruct PIN for human.

It is important to stress that when querying interactions from these resources, high-confidence should be preferred over size. In a recent study, Rolland and colleagues assembled PIs from 7 public databases and found that interactions supported by multiple sources can be validated at rates that are significantly higher than those of PIs supported by a single method (Rolland et al., 2014). This is critical, because meaningful results about human health and disease can only be achieved when using high-confidence PINs.

## 4. Protein interaction networks in health and disease

**Figure 2:**
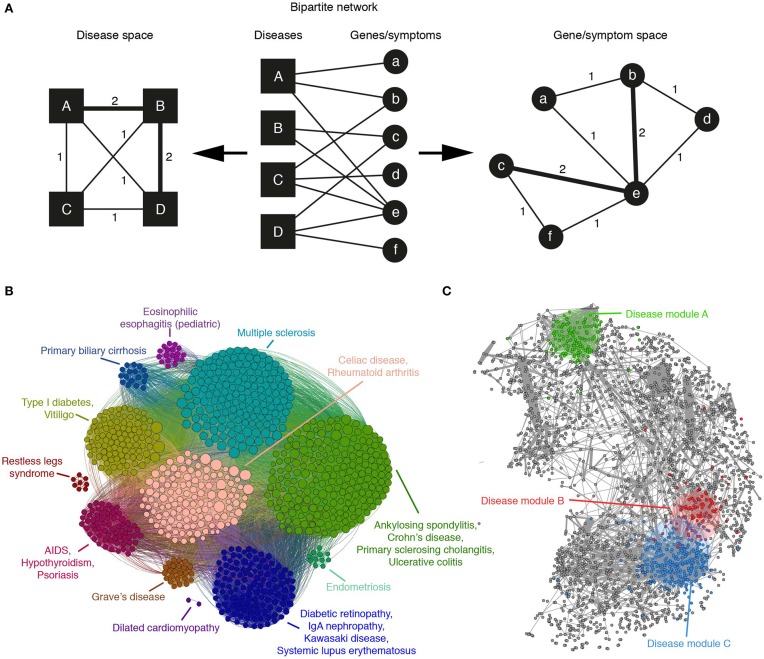
**(A)** A bipartite network of diseases and their associated genes or symptoms can be mapped to the disease or gene/symptom space by linking nodes of one type that are connected with the same nodes of the other. The weight of the edges in the resulting projection indicates the number of such common nodes. **(B)** The application of a community detection algorithm to the Autoimmune Disease Network, mapped to the gene space, reveals groups of genes associated with similar disorders and high levels of co-morbidity (adapted from Alanis-Lobato et al., 2014). **(C)** An example human protein interactome in which gene products associated with diseases A, B, and C have been labeled with different colors. According to Menche et al. (2015), the topologically closer two diseases are (like B and C), the higher the GO similarity and co-expression of their associated proteins and the higher their co-morbidity and symptom similarity.

It is possible that the first work that advocated for a systems-based approach to disease is the one by Goh et al. (2007). They take advantage of the Online Mendelian Inheritance in Man (OMIM) repository to build a bipartite network of disorders linked to their associated genes (see Figure 2A middle). Starting from this network, projections are carried out, one to the *disease space* (Figure 2A left) and the other to the *gene space* (Figure 2A right). In the disease projection, they observe a giant network component, suggesting shared genetic origins of its constituent diseases. The gene projection provides phenotypic relationship between gene pairs and presents a high overlap with a network of high-quality PIs (Goh et al., 2007). Moreover, essential human genes tend to encode hub proteins and are found to be expressed in most tissues. Whereas, disease genes are less connected and possess tissue specificity (Goh et al., 2007).

A similar analysis, focused on the gene projection, was performed considering only autoimmune diseases (Alanis-Lobato et al., 2014). After the application of a community detection algorithm, it was found that genes associated with related diseases clustered together (see Figure 2B). This community organization also revealed the presence of clusters disconnected from the main network component, suggesting that the genes forming them are disease specific.

Given a set of proteins associated with a patient's phenotype, Lage and co-workers are able to rank disease-causing proteins as the top candidates with the help of a phenotype similarity score. This also allows them to identify previously unknown disease-causing complexes (Lage et al., 2007). In a similar fashion, a tool named CIPHER scores and prioritizes phenotype-gene pairs, based on an integrated human protein and phenotype network, to reliably predict disease genes (Wu et al., 2008).

In 2014, Zhou and colleagues extracted disease and symptom terms from the Medical Subject Headings (MeSH) in PubMed and linked diseases with symptoms via bibliographic records (Figure 2A middle). Instead of simply mapping this network to the disease space, they describe each disease with a vector of symptoms, with entries quantifying the strength of association between each symptom and the disease. Later, they compute a pairwise cosine-similarity matrix between these vectors and only the most significant similarities are considered to construct a network of weighted links between diseases (Zhou et al., 2014). Analysis of the resulting network shows that disease pairs with high symptom similarity are more likely to share associated genes and PIs. This symptom-based disease network is also organized in highly interconnected communities of similar diseases, which shows that similar symptoms imply similar disorders.

The recent work of Menche and colleagues is quite relevant, as it shows that, despite its incompleteness and biases, the current human PIN can be mined and integrated with disease data to uncover pathobiological relationships between disorders and better understand their etiology. After compiling a network of roughly 140k interactions between more than 13k human proteins, nodes are labeled with their associated diseases with the help of OMIM and a set of 299 disorders defined by MeSH. Although the disease module hypothesis predicts that proteins associated with the same trait should be highly interconnected (Barabási et al., 2011; Loscalzo and Barabasi, 2011), they find that only a few disease-specific proteins form a connected subgraph. Whereas, the rest appear to be randomly distributed in the PIN because missing links isolate them from their module (Menche et al., 2015). In spite of this result, the small disease subgraphs are significantly larger than the random expectation and their topological properties are biologically meaningful: GO similarity between module members is significantly high and the topologically closer two diseases are, the higher the GO similarity and co-expression of their associated proteins and the higher their co-morbidity and symptom similarity (see Figure 2C).

## 5. Conclusion

Viewing the relationships between cell compartments and their constituting molecules as a complex circuitry of tightly interconnected components is widespread in systems biology (Vidal et al., 2011). This has led to breakthroughs that the study of the individual system components would not have made possible (Takahashi and Yamanaka, 2006; Levine and Oren, 2009; Ravasi et al., 2010). However, available interactomes are far from complete, which makes the production of high quality datasets crucial to unravel the complex relationships between genotype and phenotype (Barabási et al., 2011; Loscalzo and Barabasi, 2011).

Since the identification of biological features to distinguish between interacting and non-interacting proteins is very difficult, mining the topological characteristics of PINs is useful in the reliability assessment and prediction of PIs (Cannistraci et al., 2013b). The best candidates can be integrated with resources of high-confidence PIs to reconstruct well-grounded interactomes (Szklarczyk et al., 2015). Clinical and pathological information can then be superimposed on these networks to detect disease modules, identify co-morbidity and similarities between diseases and even make new protein-disorder associations. All of this by using simple, yet powerful network-based tools (Goh et al., 2007; Alanis-Lobato et al., 2014; Menche et al., 2015).

As the quantity and quality of molecular datasets increase, network science offers a new means to analysing interacting gene products at a systems level (Loscalzo and Barabasi, 2011). This will allow, in the near future, for a redefinition of diseases as sub-networks of a molecular interactome, overlapping with or in close proximity to other similar diseases, rendering a clear picture of the network components whose perturbation has phenotypic impact. Consequently, the integration and holistic analysis of genetic, genomic, chemical, environmental, clinical, and therapeutic data are rapidly driving the development of network medicine, a promising approach aimed at unraveling disease etiology.

### Conflict of interest statement

The author declares that the research was conducted in the absence of any commercial or financial relationships that could be construed as a potential conflict of interest.
